# Swimming induced pulmonary oedema in athletes – a systematic review and best evidence synthesis

**DOI:** 10.1186/s13102-018-0107-3

**Published:** 2018-11-01

**Authors:** Erik Hohmann, Vaida Glatt, Kevin Tetsworth

**Affiliations:** 1 Faculty of Health Sciences, University of Pretoria, Pretoria, South Africa; 2 Department of Orthopaedic Surgery and Sports Medicine, Dubai, United Arab Emirates; 3 Valiant Clinic/Houston Methodist Group, PO Box 414296, City Walk, 13th street, Dubai, United Arab Emirates; 4 University of Texas Health Science Center, San Antonio, TX USA; 5 Department of Orthopaedic Surgery, Royal Brisbane Hospital, Herston, Australia; 6 Department of Surgery, School of Medicine, University of Queensland, Brisbane, Australia; 7 Orthopaedic Research Institute of Australia, Queensland University of Technology, Brisbane, Australia

**Keywords:** Pulmonary oedema, Immersion pulmonary oedema, SIPE, Swimming, Athletes, Triathlon

## Abstract

**Background:**

Swimming induced pulmonary oedema is an uncommon occurrence and usually presents during strenuous distance swimming in cold water. The prevalence is most likely underreported and the underlying mechanisms are controversial. The purpose of this study was to summarize the evidence with regards to prevalence, pathophysiology and treatment of swimming induced pulmonary oedema in endurance athletes.

**Methods:**

Medline, Embase, Scopus and Google Scholar were searched and level I-IV from 1970 to 2017 were included. For clinical studies, only publications reporting on swimming-induced pulmonary oedema were considered. Risk of bias was assessed with the ROBINS-I tool, and the quality of evidence was assessed with the Cochrane GRADE system. For data synthesis and analysis, a best evidence synthesis was used.

**Results:**

A total of 29 studies were included (174 athletes). The most common symptom was cough, dyspnoea, froth and haemoptysis. The risk of bias for the clinical studies included 13 with moderate risk, 3 with serious, and 4 with critical. Four of the pathophysiology studies had a moderate risk, 3 a serious risk, and 1 a critical risk of bias. A best evidence analysis demonstrated a strong association between cold water immersion and in increases of CVP (central venous pressure), MPAP (mean pulmonary arterial pressure), PVR (peripheral vascular resistance) and PAWP (pulmonary arterial wedge pressure) resulting in interstitial asymptomatic oedema.

**Conclusion:**

The results of this study suggest a moderate association between water temperature and the prevalence of SIPE. The presence of the clinical symptoms cough, dyspnoea, froth and haemoptysis are strongly suggestive of SIPE during or immediately following swimming. There is only limited evidence to suggest that there are pre-existing risk factors leading to SIPE with exposure to strenuous physical activity during swimming. There is strong evidence that sudden deaths of triathletes are often associated with cardiac abnormalities.

## Introduction

The question as to whether pulmonary oedema can develop during exercise in humans remains controversial [1]. It is a well-recognised condition in racehorses, and the reported incidence ranges from 0.2 to 13% [2]. In humans, only a few clinical case reports have been published describing the condition specifically in runners, cyclists, or cross-country skiers [3–5].

Swimming induced pulmonary oedema (SIPE) was first described by Wilmshurst et al. in eleven divers with no demonstrable cardiac abnormality who had up to seven episodes when swimming or scuba diving [6]. The condition would appear to be unusual, with less than 500 cases described in the published literature [7–13]. The condition usually presents with dyspnea, cough, haemoptysis, increased sputum production, wheezing, and chest pain [7–9]. Surprisingly, more than 90% of athletes have basal inspiratory crackles on routine clinical examination during competition [9].

The pathophysiology of SIPE is not fully understood, but underlying cardiac abnormalities or dysfunction and inflammatory processes have been suggested as possible predisposing conditions [12, 14]. During exercise, the transport of oxygen across the alveolar-capillary membrane increases up to five-fold [15]. This is accompanied by an increase in pulmonary arterial and left atrial pressures [1]. In middle-aged and mature athletes these pressures are significantly higher than in healthy normal subjects [16]. Hypoxia contributes to higher pulmonary arterial pressure and further increases the likelihood of pulmonary oedema [17]. Increased pulmonary arterial pressures, combined with dramatically decreased intrathoracic pressures during inspiration, could result in exudation of fluid into the alveoli [1]. With immersion in cold water, redistribution of blood from the extremities to the thorax occurs resulting in increased blood volume in the central veins, further increasing pulmonary arterial systolic pressure [12, 18–20]. In SIPE susceptible athletes, Wilmshurst has suggested that greater vascular resistance in combination with cold water immersion results in an increase in afterload, leading to hydrostatic oedema [6].

The purpose of this systematic review was to summarize the evidence with regards to the prevalence, pathophysiology, and treatment of swimming induced pulmonary oedema in endurance athletes.

## Methods

The research was conducted according to the methods described in the Cochrane Handbook [21]. The results are reported according to the Preferred Reporting Items for Systematic Reviews and Meta-Analysis (PRISMA) guidelines statement [22].

### Eligibility criteria

Published literature was screened for prospective and retrospective level one to level four studies and case reports from 1970 to 2017 reporting on the incidence, pathophysiology, treatment and prevention of swimming induced pulmonary oedema in endurance athletes. Those identified were considered for inclusion if they reported on laboratory based and experimental studies in adult endurance athletes between the age of 18 and 60 with swimming-induced or exercise induced pulmonary oedema. For clinical studies, only publications reporting on swimming-induced pulmonary oedema were considered. Studies were excluded if the study involved scuba divers, military or other professional divers, and case reports and experimental studies investigating the effect of full body immersion simulating diving activities. If studies reported on both swimming and diving induced pulmonary oedema, the abstract and full text was screened and the cases related to swimming extracted. Conference proceedings or abstracts, expert opinions (level V) and review articles were excluded. It is acknowledged that the omission of grey data could potentially result in publication bias, but will also substantially reduce selection bias.

### Information sources and literature research

A systematic review of the literature utilizing Medline, Embase, Scopus and Google Scholar was performed to identify all publications in the English and German literature related to swimming and exercise induced pulmonary oedema. The following search terms and Boolean operators were used: “exercise” AND/OR “swimming” AND/OR “induced” AND/OR “pulmonary oedema”; “endurance athlete” AND/OR “triathlete” AND/OR “pathophysiology”; AND/OR “physiology and pathology”. The search was performed on 20 November 2017, and two reviewers independently assessed the titles and abstracts. All eligible articles were then manually cross-referenced to ensure any other potential studies were included. Disagreements were resolved by consensus; if no consensus was achieved a third independent researcher was consulted and the difference resolved by majority vote.

### Data extraction and quality assessment

For studies that met the inclusion criteria, an electronic data extraction form was used to obtain the following information from each article: author, journal and year of publication, any conflicts of interest, water temperature, symptoms, outcomes, and physiological data collected by the authors. Two authors independently completed data extraction, and the third reviewer and senior author verified the data.

Risk of bias for randomized-control level one and two studies was assessed adapting the Cochrane Collaboration’s Risk of Bias Tool [21]. Because the Cochrane Handbook does not specifically describe the assessment of risk of bias for observational and laboratory studies, the ROBINS-I tool for the assessment of bias in observational studies was utilized [23]. Briefly, the following domains of bias were considered: due to confounding, selection bias, bias in classification of interventions, bias due to deviations from intended interventions, bias due to missing data, bias related to measurement of outcome, and bias in the selection of the reported results. Each domain of bias is then evaluated with one of the following responses: “yes”, “probably yes”, “probably no”, and “no”, with probable responses having similar implications as “yes” or “no”. The categories of judgement for each study are low, moderate, serious, and critical risk of bias [23].

The senior author assessed the quality of evidence for each applicable outcome measure using the GRADE system, and the second reviewer verified these assessments [21]. The GRADE assessment defines the quality of evidence for observational studies as low, but allows either down- or upgrading based on various factors. Studies were downgraded if limitations in the design, indirectness of evidence, unexplained heterogeneity, imprecision of results, and high probability of publication bias were observed, and study quality was therefore reduced from low to very low. Studies were upgraded if there was a large magnitude of effect, if all plausible confounding would reduce a demonstrated effect or suggest a spurious effect when results showed no effect, and if there was a dose-response gradient observed. All institutional and author information was concealed to the second reviewer to reduce reviewer bias. Any disagreement between reviewers was resolved by consensus, or by arbitration between the two senior authors.

### Data synthesis and analysis

Data pooling and meta-analysis was deemed not possible by the authors because of study heterogeneity, and the fact that none of the included studies were level I or II evidence. A best evidence synthesis, as suggested by Slavin, was therefore utilised [24]. Based on the guidelines for systematic reviews in the Cochrane Collaboration [25], the ranking of the levels of evidence developed by van Meer et al. [26] was used. Strong evidence was defined as two or more studies with low risk of bias, and with > 75% of the studies reporting consistent finding. Moderate evidence was defined as one low risk of bias study and two or more high risk of bias studies, with consistent findings in more than 75% of the included studies. Limited evidence was defined as one or more high risk of bias studies or one low risk of bias study, with > 75% of the studies reporting consistent findings. Conflicting evidence was defined as contradictory findings, with less than 75% of the studies reporting consistent findings. No evidence was defined if none of the included studies provided any evidence.

## Results

### Study selection and characteristics

**Fig. 1:**
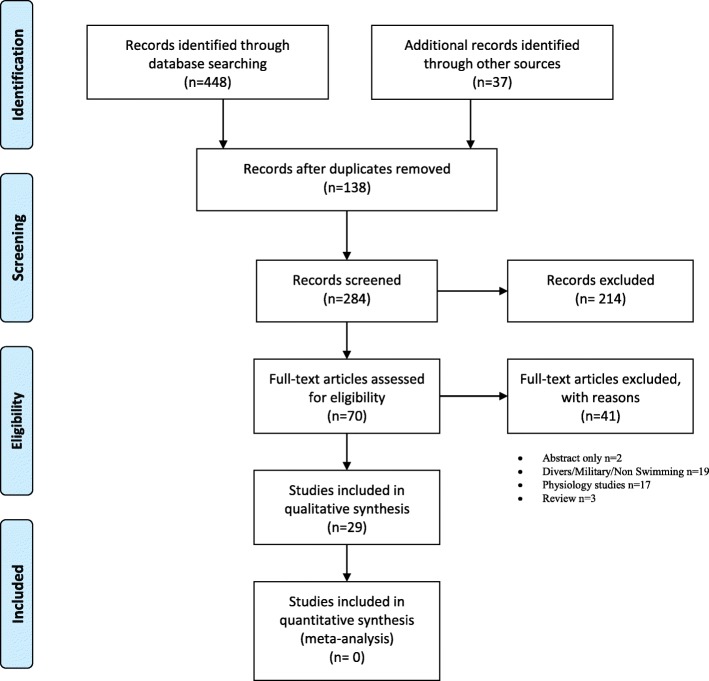
PRISMA Flow Diagram: 485 studies between the years 1970 and 2017 were identified. Two hundred and eighty four studies were screened and 214 excluded after reviewing their abstracts. For the remaining 70 studies, examination of the full text manuscript was conducted but only 29 articles met the eligibility criteria

The literature search identified 485 studies between the years 1970 and 2017 for consideration. Of these, 284 studies were screened and 214 excluded after reviewing their abstracts. For the remaining 70 studies, examination of the full text manuscript was conducted. Only 29 articles met the eligibility criteria and were included in the qualitative synthesis (Fig. 1). Nineteen of the 29 studies were case studies [9, 10, 12, 13, 27–41]. One study described diagnostic techniques [42], while nine articles [7, 8, 14, 18, 43–47] investigated pathophysiology, and one of these studies [7] also suggested treatment options. The study characteristics and results are summarized in Tables 1 and 2.

**Table 1:** Study characteristics and outcomes of the clinical studies

	Cases	gender	age	Water temperature	symptoms	outcome
Pons 1995	4			18-21 °C	Dyspnoea (2), cough (4), froth (4)	Resolved within 1 h (3), 8 h (1)
Weiler 1995	8	M = 8	18	23 °C	Cough (7), haemoptysis (8)	Resolved (6) recurring (2)
Roeggla 1996	1	F = 1	54	14 °C	Dyspnoea, cough, froth, haemoptysis	Resolved
Lund 2003	3	M = 3	24.3	19 °C	Dyspnoea (3), cough (3), confusion (2), blurred vision (1), froth (1)	Resolved within 48 h
Adir 2004	70	M = 70	18–19	16-22 °C	Dyspnoea (70), cough (67), haemoptysis (39), froth (63),	Resolved within 24 h, recurrence (16)
Biswas 2004	1	M = 1	36	< 22 °C	Dyspnoea, cough, froth	resolved
Shuppak 2004	21	M = 21	18–19	16-18 °C	Dyspnoea, cough, froth, haemoptysis	Resolved (18), recurring (3)
Wilmshurst 2004	1	M = 1	35	13 °C	Dyspnoea, haemoptysis, froth	recurrence
Deady 2006	1	F = 1	38	15 °C	Dyspnoea, haemoptysis	Resolved within 24 h
Beinhart 2007	1	F = 1	54	22 °C	dyspnoea	resolved
Wenger 2007	1	M = 1	43	20 °C	Dyspnoea, froth, chest pain	Resolved within 24 h
Noti 2009	1	F = 1	23		Dyspnoea, froth	Resolved within 24 h
Carter 2011	3	F = 3	49.7	15-19 °C	Dyspnoea (3), froth (2), cough (1), haemoptysis (1), chest pain (2)	Resolved (1), Recurrence (1)
Ma 2013	5	M = 5	42.6	< 22 °C	Dyspnoea (3), cough (2), Haempotysis (3), froth (1)	Resolved within 1 week
North 2013	1	F = 1	26	< 22 °C	Dyspnoea, cough, froth	Resolved within 48 h
Casey 2014	2	M = 1, f = 1	57.5	< 22 °C	Dyspnoea (2), froth (1), cough (1), chest pain (1)	Resolved within 24 h
Peacher 2015	41		50.1			
Yamamashi 2015	5	M = 1	41.8	21 °C	Dyspnoea (5), haemoptysis (1), cough (1)	Resolved within 10 days
Beale 2016	4	M = 4	36.7		Dyspnoea (4), cough (3), haemoptysis (2), froth (3)	Resolved (4), recurrence (2)
*N* = 20	174	M = 165F = 9		13-22 °C	Dyspnoea (90), cough (97), froth (89), haemoptysis (58), blurred vision (1), chest pain (3)	Resolved (185), recurrence (25)

**Table 2:** Study characteristics and outcomes of the clinical studies

Caillaud 1995	Pulmonary function	DL_CO_, K_CO,_ V_A_, Pulmonary Volume (CT), MLD	Pre- and post Olympic Triathlon Water temperature 14 °C	No change in VC and TLC, DLCO and KCO reduced, slight increase in extrapulmonary water, significant increase in MLD
Choi 2004	Arrythmia Long QT syndrome		Genetic testing	LQTS +CPVT1 gene present in 90.7% swimming triggered events
Ludwig 2006	Pulmonary function	TLC, VC, FEV_1_, FEV_1_/FVC, DL_CO_	Non randomized study, testing following cycle ergometry, SIPE subjects versus control group	No difference between groups
Wester 2009	Pulmonary function	VT, V_O2_, VE, VD/VT, V_CO2_, Ph, P_CO2_, P_O2,_ MAP, MPAP, PAWP, CVP	Dry and immersion exercise in cold (19 °C) and warm (28 °C)	MPAP, CVP higher in cold water
Fraser 2010	Pulmonary function	VC, FEV_1_, FEV_25–75_, HCVR, VO_2max_, PAP, MAP, MPAP, CVP	Cold water immersions (20-21 °C) Hyper vs normoxia	Cold water immersion associated with higher mean PAP, PVR, MPAP favouring conditions for pulmonary oedema, PAWP approached threshold to pulmonary capillary leak pressures
Harris 2010	Deaths in Triathlons	Heart Morphology	Autopsy (n = 9)	7 left VH, 1 congenital coronary artery anomaly
Pingitore 2011	Echocardiography, Chest Ultrasound, Pulmonary function	VC, FEV1, Troponin, IL LV systolic function and volume	Pre- and post Ironman event	Transient and interstitial pulmonary oedema in all athletes
Moon 2016	Deaths in Triathlons	Heart mass, ventricular thickness, coronary abnormalities	Autopsy (*n* = 23)	95% heart mass > than normal, 23 > 70% CAD narrowing, 32% > 50%CAD narrowing
Moon 2016	Pulmonary function	CVP, MAP, MPAP, PAWP	Submersion in 20 °C water	CVP 23% increase, PAWP 25% increase, MPAP 26% increase, MAP 7% increase
Shearer 2009	Brain natriuretic peptide (BNP)	BNP	BNP levels in 6 cases confirmed with SIPE in the ER	Within normal for all subjects

### Risk of bias

The findings of the risk assessment for bias are summarized in Tables 3 and 4. None of the eligible studies included randomized clinical trials, and risk of bias for all manuscripts was therefore assessed using the ROBINS-I tool [23]. Nineteen of the 29 included studies were case series [9, 10, 12, 13, 27–41], one study described diagnostic techniques [42], nine articles [7, 8, 14, 18, 43–47] investigated pathophysiology, and one study suggested treatment options [7]. Twelve of the 19 case series reporting on prevalence were assessed to have a moderate risk of bias [10, 27–30, 32–36, 38, 39]. Three studies had a serious risk of bias [13, 31, 41]. Four of the studies were considered to have a critical risk of bias [9, 12, 37, 40]. Confounding, selection, intervention, and measurement bias was present in the study by Adir et al. [9]. The authors utilized 70 participants who were enrolled in a swimming program, yet spirometry was only performed in a subgroup of 37 participants [9]. Definite confounding and selection bias, and probable bias for the other five parameters, were found in Ma &Dutch small case series [37]. SIPE was diagnosed in five triathletes at different stages of the competition, while three athletes required hospital admission [37]. Selection bias and probable classification, missing data, and measurement bias was observed in the case series from Peacher et al. [12]. Volunteers were recruited from a previous physiological study, while the history and SIPE diagnosis was self-reported and the presented data for all identified volunteers was incomplete [12]. Similar to Ma & Dutch [37], Yamamashi et al. [40] have reported on six athletes with a diagnosis of SIPE during a triathlon, and bias was present for all seven items.

**Table 3:** Risk assessment for bias for the clinical studies

	Confounding Bias	Selection Bias	Classification Bias	Intervention Bias	Missing Data Bias	Measurement Bias	Reporting Bias	Bias
Pons 1995	n	py	pn	pn	n	py	n	moderate
Weiler 1995	py	py	n	n	n	n	n	moderate
Roeggla 1996	n	n	n	n	n	n	n	moderate
Lund 2003	pn	py	n	n	n	py	pn	moderate
Adir 2004	y	y	n	y	n	y	pn	critical
Biswas 2004	n	n	n	n	n	n	n	moderate
Shuppak 2004	y	y	n	n	n	py	py	serious
Wilmshurst 2004	n	y	n	n	y	n	n	serious
Deady 2006	n	n	n	n	n	n	n	moderate
Beinhart 2007	n	n	n	n	n	n	n	moderate
Wenger 2007	n	n	n	n	n	n	n	moderate
Noti 2009	n	n	n	n	n	n	n	moderate
Carter 2011	n	n	n	n	n	n	n	moderate
Ma 2013	n	py	py	pn	n	y	py	critical
North 2013	n	n	n	n	py	py	n	moderate
Casey 2014	n	n	n	n	n	n	n	moderate
Peacher 2015	pn	y	py	n	py	py	pn	critical
Yamamashi 2015	y	y	py	py	py	py	py	critical
Beale 2016	n	y	pn	py	pn	py	pn	serious
North 2013

**Table 4:** Risk assessment for bias the basic science studies

	Confounding Bias	Selection Bias	Classification Bias	Intervention Bias	Missing Data Bias	Measurement Bias	Reporting Bias	Bias
Pathophysiology								
1995 Caillard	n	n	n	n	n	n	n	moderate
2004 Choi	py	py	pn	pn	py	n	py	critical
2006 Ludwig	py	py	n	n	n	n	n	serious
2009 Wester	n	n	n	n	n	n	n	moderate
2010 Harris	n	py	n	n	y	n	py	serious
2010 Fraser	n	n	n	n	n	n	n	moderate
2011 Pingitore	n	n	n	n	n	n	n	moderate
2016 Moon	y	y	n	n	n	n	n	serious
DX								
2009 Shearer	y	py	pn	pn	pn	n	n	serious
RX								
2016.1 Moon	n	n	n	n	n	n	n	moderate

Four of the studies reporting on pathophysiology had a moderate risk of bias [18, 43, 45, 47], three studies a serious risk of bias [8, 14, 46], and one study [44] a critical risk of bias. Probable confounding, selection, missing data, and reporting bias were determined to be present in the study by Choi et al. [44]. They performed genetic testing for long QT syndrome in 388 unrelated patients, and also evaluated the family history by review of medical records or phone interview. A specific gene was identified in only two thirds of swimming related cases. Shearer and Mahon [42] reported on the value of brain natriuretic peptide (BNP) to diagnose SIPE, but only six cases were tested and no conclusion could be drawn. The risk of bias in this study was assessed as serious [42]. There was only one publication discussing treatment options, by Moon et al., who reported on the benefits of sildenafil in athletes with a history of SIPE [7]. Utilizing radial artery and pulmonary artery catheters in 10 volunteers performing immersion cycle ergometry, significant differences between SIPE susceptible subjects and a control group were observed. Although these differences were not observed following administration of 50 mg Sildenafil, the risk of bias was assessed as moderate.

Utilizing the GRADE quality scheme, none of the included studies were upgraded. Of the 19 prevalence and case series studies, ten were downgraded to very low quality because of limitations in the design and imprecision [9, 10, 12, 13, 27, 28, 31, 37, 40, 41]. Of the ten studies investigating pathophysiology, three were downgraded to very low quality based on design limitations [7, 14, 42, 44].

### Prevalence

The prevalence of SIPE in humans is not clear and possibly underreported. Pons et al. reported a prevalence of 1.1% in a mixed cohort of swimmers and divers, and Miller et al. estimated a population prevalence of 1.4% in triathletes [27, 48]. A prevalence of 1.8% has been reported previously in combat military swimmers [9]. The pooled data here from the 19 included studies reported a total of 174 athletes; 165 were males and 9 were females (Table 1). The most common symptom was cough (*n* = 97), dyspnoea (*n* = 90), froth (89) and haemoptysis (*n* = 58). Uncommon symptoms included chest pain (*n* = 3) and blurred vision. Chest pain was only reported by athletes older than 40 years. In all but 5 athletes symptoms resolved within 48 h [40]. Recurrence of SIPE was reported in 25 athletes (14.4%). Reported risk factors included asthma (*n* = 2), environmental allergies (n = 2), hypertension (*n* = 1), previous pneumonia (n = 1), type 1 diabetes mellitus (n = 1), and a history of smoking (n = 1). The water temperature was lower than 23 °C in all cases and ranged from 13 to 23 degrees Celsius. The best evidence-based analysis suggests that there was moderate indication for an association between water temperature and the prevalence of SIPE. The water temperature resulting in SIPE in the included clinical studies ranged from 13 [31] to 23 degrees Celsius [28]. In the studies investigating pathophysiological variables the temperatures ranged from 14 [43] to 21 degrees Celsius [45].

Further best evidence analysis demonstrated that the presence of the clinical symptoms of cough, dyspnoea, froth, and haemoptysis are all strongly associated with SIPE in athletes during or immediately following swimming events or during the swimming leg during triathlons. There is limited evidence to suggest that there are pre-existing risk factors leading to SIPE with exposure to strenuous physical activity during swimming.

### Pathophysiology

Of the eight studies included here, six investigated pulmonary function in swimmers and triathletes [7, 14, 18, 43, 45, 47], two investigated deaths in triathlons [8, 46], and one was related to cardiac factors [47]. Cold water swimming resulted in an increase in CVP, MPAP, mean PVR, and PAWP. A significant reduction in DLCO and KCO was noticed by Caillaud et al. [43], which was associated with a significant increase in MLD and an increase in extra-pulmonary water. Pingitore et al. reported transient interstitial pulmonary oedema in all athletes diagnosed with SIPE [47]. In contrast, Ludwig et al. could not demonstrate any differences in pulmonary function parameters between a group of athletes with SIPE and a control group without [14]. Cardiac abnormalities were found in more than 90% of deaths related to the triathlon swim leg [8, 46]. Harris reported that 7/9 athletes had left ventricular hypertrophy, and 1/9 a congenital coronary artery anomaly [46]. Moon demonstrated that 95% had a larger heart mass than normal, 23% showed coronary artery stenosis of > 70%, and 32% showed coronary artery stenosis of > 50%. (Table 2) [8].

The only study investigating cardiac parameters reported significant decreases of LV end-diastolic volumes, significant decreases in the ejection fraction from 72 to 66%, a decrease in right heart function, and significant increases in cardiac troponin from 0.02 to 0.14 ng/ml following an Ironman race [47], while also demonstrating significant increases in BNP. They concluded these exercise induced haemodynamic changes were the cause of the transient and asymptomatic exercise induced interstitial pulmonary oedema observed in all 31 athletes included in their study [47].

Best evidence analysis suggests there was strong evidence that cold water immersion results in increases for CVP, MPAP, PVR and PAWP, leading to an increase in asymptomatic interstitial oedema. There is also strong post-mortem evidence from triathletes having died during events that pathological findings are often associated with coronary artery stenosis and left ventricular hypertrophy.

## Discussion

The results of this best evidence analysis suggest that swimming in cold water is one of the potential triggers for SIPE. Cold water immersion increases CVP, MPAP, PVR and PAWP, providing a possible explanation why individuals become susceptible to interstitial pulmonary oedema. The reported water temperature in the included studies investigating cold water induced SIPE ranged from 13 to 23 degrees. Based on these reports, cold water could therefore be defined as a water temperature of less than 23 degrees Celsius. The index of suspicion for SIPE should be high when athletes present with cough, dyspnoea, froth, and haemoptysis. Best evidence analysis could not identify specific risk factors leading to pulmonary oedema with exposure to swimming in cold water.

Currently there is no evidence as to why certain individuals are susceptible to SIPE. Miller et al. conducted a review of the 104,887 Triathlon USA members via an online survey [48]. They reported a prevalence of 1.4%, indicating that very few athletes are affected. However, the validity of their findings are severely limited as only 1400 members responded to the survey. For this study, the definition of SIPE was limited to a single variable: ‘cough productive of pink frothy or blood-tinged secretions’. In addition to the very low response rate, it is highly likely that milder cases of SIPE were missed in this survey. In a small comparative study, Moon et al. suggested that an exaggerated increase in MPAP and PAWP could be a possible aetiology for SIPE [7]. In subjects with a confirmed history of SIPE, the authors demonstrated lower baseline oxygen consumption, heart rates, stroke volumes, and cardiac output [7].

Earlier, Caillaud et al. used CT scanning in eight male athletes to measure water accumulation in the lungs before and after a triathlon [43]. They demonstrated an increased mean lung density following the race, suggesting interstitial fluid accumulation. McKenzie et al. used MR imaging to investigate SIPE, and showed a 9.4% increase in pulmonary extravascular water [49]. Miles et al. described a significant increase in alveolar-capillary membrane resistance that was associated with a decrease in DLCO and DM and remaining vital capacity after a marathon race [50]. They interpreted these findings as the presence of subclinical pulmonary oedema. Six studies investigated pulmonary function in swimmers and triathletes [7, 14, 18, 43, 45, 47]. They reported cold-water swimming resulted in an increase in CVP, MPAP, mean PVR, and PAWP, and also a significant reduction in DLCO and KCO [7, 14, 18, 43, 45, 47]. Unfortunately, none of these authors differentiated between the potential causes and consequences of SIPE. Increases in PAP, PVR and PAWP are the likely causes of SIPE, whereas reductions in DLCO and KCO as a result of secondary increased resistance at the alveolar-capillary interface are likely consequences of SIPE. Similar to SIPE, high altitude pulmonary oedema (HAPE) may have very similar pathophysiological changes. Eldridge et al. investigated the effect of high altitude on pulmonary blood flow and alveolar leakage in eight endurance athletes [51] Using cycle ergometry with 90% maximal effort, all athletes developed permeability oedema even at normal altitude. Increased altitude resulted in significantly higher fluid leakage into the alveolar space, a typical pathophysiological response to exercise under hypobaric conditions. Cold water immersion and the physiological responses to race conditions such as nervousness, anxiety, and excitement may trigger exaggerated sympathetic responses, resulting in SIPE in susceptible individuals [52, 53].

However, the hypothesis of exercise induced interstitial oedema is controversial, and other researchers have shown that exercise does not result in pulmonary oedema [54, 55]. Similar to Cailleaud et al. [43], Manier et al. [55] examined lung density using CT scans. In nine trained endurance runners before and after a 2 h treadmill run, they could not demonstrate any significant post exercise changes in lung mass or density. Hodges et al. examined extravascular lung water in 10 male subjects undergoing 60 min of cycling in both hypoxic and normoxic conditions [54]. They could not demonstrate any evidence of post-exercise pulmonary oedema under either condition.

Whilst best evidence analysis could not identify specific risk factors, Peacher et al. reported that of the 36 athletes with immersion pulmonary oedema, 24 (67%) had pre-existing cardiac or pulmonary abnormalities, compared to only 45% in a historical control group [12]. These abnormalities included hypertension, left ventricular hypertrophy, cardiomegaly, chronic atrial fibrillation, coronary artery disease, asthma, and exercise induced cough [12]. However, the validity of their findings is severely limited due to incomplete medical records and historical controls. Furthermore, the authors included divers, introducing further selection bias. In a case-control group, Miller et al. compared 1411 athletes with SIPE to a group of 31 healthy control subjects. Advanced age (OR 3.35), hypertension (OR 4.87), diabetes (OR 7.63), fish oil use (OR 3.1), and a long course (OR 3.32) were all associated with higher risk [48]. The value of this study is again limited by the low response rate, the retrospective survey design, and the self-reported diagnoses.

Swimming is one of the three disciplines during a triathlon, and respiratory symptoms are most often reported during the swim leg [7, 8]. Moon et al. and Harris et al. investigated the causes of death during the triathlon swim leg [8, 46]. SIPE was reported to be the most likely cause, and the reasons for pulmonary oedema in these athletes was explained by the presence of left ventricular hypertrophy, lusitropy, abnormal conduction, and coronary artery stenosis exceeding 50% resulting in possible abnormal LV diastolic compliance [8, 46]. Moon et al. reported that the majority of athletes who died had left ventricular hypertrophy [46]. However, these findings could also be related to normal physiological changes in endurance athletes. Douglas et al. determined that left ventricular wall thickness of more than 13 mm in athletes represents a pathological condition [56]. Moon reported the mean LV thickness in his series exceeded these values by two millimetres, and the observed hypertrophy was therefore most likely pathological [7, 8]. In addition, the presence of > 70% coronary artery stenosis was noted in 23%,of athletes, and > 50% in 32% of athletes, suggesting asymptomatic coronary disease may be a contributing factor [7, 8].

Harris reported one case (7%) with Wolff-Parkinson-White syndrome and two (14%) with a structurally abnormal heart [46]. Whether arrhythmias has contributed to death in these two cases cannot reliably be established but the combination of abnormal heart morphology, physiological stress reactions, and cold water immersion could certainly cause arrhythmias resulting in sudden death. Claessens et al. has demonstrated a significantly higher rate of premature ventricular beats in triathletes [57]. These findings are supported by Fuchs et al., who demonstrated athletes engaged in competitive sports develop ventricular arrhythmias during exercise [58]. Shattock and Tipton proposed autonomic conflict as a possible cause of cardiac arrhythmias during cold water submersion [53]. The activation of a sympathetic system-driven tachycardia with cold water submersion and breath holding promoting a parasympathetic system-driven bradycardia may cause arrhythmias, which can cause sudden death in vulnerable individuals [53]. Warburton et al. used signal-averaged electrocardiography (SAECG) to determine late potentials (LP) [59]. They suggested the presence of LPs may indicate that prolonged, strenuous exercise could result in electrical instability triggering arrhythmias [59].

Best evidence analysis suggested that the evidence was strong to support the association of death and cardiac pathology. The results of this study therefore strongly suggest pre-screening for middle-aged triathlon or endurance athletes as a preventative risk reduction measure. In addition, recommendations to reduce the size of the starting groups, larger wave intervals, rolling starts, allowing pre-swims to adjust to the water temperature, and well planned emergency action plans may improve swim safety [52].

Best evidence synthesis allows detailed analysis of study flaws and study characteristics when data pooling and meta-analysis is not possible. The limitations of this study are inherent to the limitations of the included studies. Seven of the 19 clinical studies had serious and critical risk of bias, and ten were downgraded with regard to study quality. Of the ten studies investigating pathophysiology, four were found to have serious and critical risk of bias, and these four studies were downgraded to very low quality. The low quality of the included studies limits both internal and external validity. It could be argued that the results of this study should therefore be viewed with caution. However, despite moderate evidence for the association between cold water immersion and the prevalence of SIPE, the findings of all 19 studies are consistent, strengthening the conclusions. Given the limited evidence between SIPE and pre-existing risk factors, further studies are needed and the evidence here must be viewed with caution. Exercise physiology and pathophysiological responses may differ between different races and people with differing ethnic origins. Of the 29 studies included in this systematic review, 28 were performed in Western countries, with populations of predominantly Caucasian origin. It is possible that the majority of the included patients were therefore also of Caucasian descent, introducing minor selection bias. However, almost all of these studies were based in the United Kingdom, USA, Canada, or Australia. These societies are all multicultural and there is a distinct possibility that individuals from a different genetic heritage were also included, although it is not possible to determine how this may, or may not, have influenced the results. Wet-bulb temperature was not reported in the included studies, although wet-bulb temperature may better reflect an individual’s perception of water temperature. However, there is no current evidence to support this possibility, and the available literature does not allow further comment regarding any potential relationship between conditions in the water and the ambient environment.

## Conclusion

The results of this best evidence systematic review suggest a moderate association between water temperature and the prevalence of SIPE. The presence of specific clinical symptoms including cough, dyspnoea, froth, and haemoptysis are strongly suggestive of SIPE during or immediately following swimming. There is only limited evidence to suggest that there are pre-existing risk factors leading to SIPE with exposure to strenuous physical activity during swimming. There is strong evidence that sudden deaths of triathletes are often associated with cardiac abnormalities.

## Abbreviations

CT: Computer tomogram
CVP: Central venous pressure
DLCO: Carbon Monoxide Diffusing Capacity
KCO: Lung transfer coefficient
MLD: Mean lung dose
MPAP: Mean pulmonary arterial pressure
PAWP: Pulmonary arterial wedge pressure
PVR: Peripheral vascular resistance
SIPE: Swimming induced pulmonary oedema
